# Differences in Pigment Content and Expression of Cocoon Color Formation-Related Genes in Multiple Silkworm Strains

**DOI:** 10.3390/insects17040435

**Published:** 2026-04-17

**Authors:** Lin Zhu, Mengli Li, Zijian Huang, Yuyang Wu, Guodong Zhao, Heying Qian

**Affiliations:** 1 Jiangsu Key Laboratory of Sericultural and Animal Biotechnology, School of Biotechnology, Jiangsu University of Science and Technology, Zhenjiang 212100, China; 2 Key Laboratory of Silkworm and Mulberry Genetic Improvement, Sericultural Scientific Research Center, Chinese Academy of Agricultural Sciences, Ministry of Agriculture and Rural Affairs, Zhenjiang 212100, China

**Keywords:** *Bombyx mori*, natural-colored cocoon, carotenoid, flavonoid, structural variation

## Abstract

Deciphering the coloration mechanism of natural-colored cocoons in the domestic silkworm (*Bombyx mori*) is of great importance for the green and sustainable development of the sericulture industry. In this study, 14 silkworm strains were selected for studying differences in the coloration mechanism of diverse cocoon colors, and the present research carried out integrated investigations from three aspects: pigment content, differences in gene expression levels, and gene structural variation. The results demonstrated that pigment accumulation presented distinct tissue-specific and strain-specific characteristics. The middle silk gland (MSG) acts as the primary locus for pigment deposition: silkworm strains forming yellow or red cocoons accumulate carotenoids at high levels in this tissue, whereas those producing green cocoons show abundant flavonoid enrichment here. Analysis of gene expression profiles indicated that the expression patterns of core transporter genes are highly correlated with the spatial distribution of pigments. The expression level of *CBP* gene in the MSG is over 10-fold higher than that in the midgut (MG) among yellow/red cocoon strains. The pivotal glycosylation gene *UGT86* displayed remarkably elevated expression in the MSG relative to other tissues across all green cocoon silkworm strains. The *CBP* gene acts as a core regulatory factor governing the transport of carotenoid pigments, and notable disparities existed in the coding region of the gene among silkworm strains with different cocoon colors. In contrast to yellow and red cocoon strains, the transcription start site of *CBP* gene is displaced in silkworm varieties that form green or white cocoons. In summary, this study clarified the expression patterns and variations in key pigment deposition-related genes at the population level for the first time and provided data references for the study of the biological basis and coloration mechanism of diverse cocoon colors.

## Simple Summary

To clarify the biological basis and coloration mechanism of diverse cocoon colors, this study compared the differences in pigment content and expression of cocoon color formation-related genes in 14 different silkworm strains. The results showed that the middle silk gland is the key site for color pigment accumulation, and special genes involved in pigment formation and transport act differently or have structural variations in multiple silkworm strains with different cocoon colors. The *CBP* gene acts as a core regulatory factor governing the transport of carotenoid pigments to the silk gland, and notable disparities existed in the coding region of the gene among silkworm strains with different cocoon colors. In contrast to yellow and red cocoon strains, the transcription start site of the *CBP* gene is displaced in silkworm varieties that form green or white cocoons. This finding holds significant social value as it provides data references for breeding silkworms that can produce natural-colored silk, which helps promote the development of eco-friendly sericulture and reduce pollution in the textile industry.

## 1. Introduction

As a typical model organism of lepidopteran insects, the domestic silkworm (*Bombyx mori*) possesses considerable economic significance in China as well as in other regions across the globe [1]. The domestic silkworm experiences four markedly different developmental phases throughout its life cycle, namely the egg, larva, pupa and moth stages, each of which features unique morphological traits and physiological properties, making it a classic representative of insects with complete metamorphosis [2]. With the advantages of easy cultivation, a short life cycle, a clear genetic background and a body size suitable for experimental operation, the silkworm has long been recognized as an ideal research model in the fields of insect genetics and molecular biology [3]. As a time-honored natural protein fiber, silk has long taken white cocoon silk as the primary raw material in the traditional textile manufacturing industry [4,5]. Yet the subsequent chemical dyeing procedures involved in silk processing are prone to causing severe environmental contamination, which has in turn made natural-colored silk an object of increasing interest for professionals in both industrial production and academic research fields [6].

In comparison with white cocoon silk, natural-colored silk not only boasts distinctive color tones, but also features a more loose internal fiber structure in most cases, which endows it with better moisture absorption and air permeability performance [7]. What is more, the natural pigments abundant in colored silk fibers, such as flavonoid compounds and carotenoids, add multiple functional characteristics to the silk, including prominent antibacterial, antioxidant and anti-ultraviolet effects [8,9,10]. These inherent pigments make the subsequent chemical dyeing and finishing processes unnecessary, which not only alleviates environmental pollution but also reduces the potential risk of chemical residue in textile products [6]. Such advantages are highly consistent with the current pursuit of green and sustainable consumption concepts in society. For these reasons, natural-colored silk has now become a research hotspot in the disciplines of sericultural science and textile material engineering.

Based on the types of pigments contained in silk, naturally colored silkworm cocoons are divided into two major categories: the yellow/red cocoon strains and the green cocoon strains [11]. The yellow/red cocoon strains display a rich spectrum of color shades, ranging from pale yellow and golden yellow to flesh color, pink and bright red. The color formation of these cocoons is mainly dependent on carotenoid substances obtained from mulberry leaves, such as α-carotene and β-carotene [12]. As an insect entirely dependent on exogenous pigments, the silkworm cannot synthesize pigment compounds on its own and must obtain them by feeding on mulberry leaves [13]. Studies have shown that carotenoids from mulberry leaves, such as α-carotene, β-carotene, and violaxanthin, are absorbed by MG epithelial cells, transported via the hemolymph to the silk gland, and finally bind with sericin proteins to form the cocoon color [14]. This process is regulated by multiple genes, for instance, the *Y* gene controls the entry of carotenoids from the MG into the hemolymph, the *C* gene regulates pigment transport from the hemolymph to the silk gland, and the *I* gene suppresses transmembrane pigment transport [15]. Molecular studies indicate that the carotenoid-binding protein (CBP) encoded by the *Y* gene (*CBP* gene, NCBI ID. 692547) and the Cameo2 protein encoded by the *C* gene function cooperatively, participating in the specific recognition and transport of carotenoids in the MG and silk gland, respectively. Additionally, SCRB15, the product of the *F* gene (flesh-colored cocoon gene), likely mediates the enrichment of β-carotene in the silk gland [16]. Structural variations in these genes (such as the insertion of introns and deletion of exons), together with the differences in their expression patterns, act as the core factors that drive the diverse color phenotypes exhibited by the yellow/red cocoon silkworm strains [11].

Green cocoon silkworm strains are mainly characterized by two color tones of light green and deep green, and their cocoon color formation is mainly dependent on flavonoid substances [17]. Silkworms obtain flavonoids through ingesting mulberry leaves, and these compounds undergo intracellular modifications like glycosylation before being integrated into sericin components [18]. Relevant studies have shown that the flavonoid content in the cocoon layer of green cocoon varieties is remarkably higher than that of white and yellow cocoon varieties, and the depth of green cocoon color presents a positive correlation with the cumulative content of flavonoids [19]. The uridine diphosphate glycosyltransferase (UGT) gene family, which includes *UGT86* (*UGT10286* gene, NCBI ID. 100500754) and *UGT89* among its members, exerts a pivotal effect on the glycosylation modification of flavonoids, and the expression of these genes shows obvious tissue-specific and variety-specific characteristics [20]. For instance, *UGT8*6 is highly expressed in the silk gland of green cocoon strains, whereas *UGT30* and *UGT59* may participate in the synergistic regulation of flavonoid transport processes between the midgut and silk gland. In addition, flavonoid compounds accumulate in large quantities in silkworm pupae, with their content being several times that in the cocoon layer, which implies that the pupa may act as a metabolic reservoir for pigment substances in silkworms [21].

These natural pigments not only serve as the core contributors to silkworm cocoon coloration but also carry important biological functions in the silkworm body. Carotenoids and flavonoids both act as potent antioxidants, which can effectively scavenge free radicals, mitigate oxidative stress in organisms, and take part in the regulatory processes of immune responses and reproductive performance [22]. For example, in avian species, the deposition of carotenoids in eggs can facilitate embryonic development and improve hatching success rates, a biological phenomenon that shares evolutionary conservation with the pigment transport mechanisms found in silkworms [23]. In addition, the antibacterial activity and ultraviolet resistance of flavonoid compounds also lay a solid theoretical foundation for the development and functional application of naturally colored silk materials.

While the functions of core genes associated with silkworm cocoon coloration have been relatively well elucidated, it remains unconfirmed through systematic multi-strain research whether these genes and the metabolic pathways they regulate display consistent expression and variation patterns across silkworm varieties with distinct genetic backgrounds. To resolve this scientific question, this study selected 14 silkworm strains with divergent cocoon colors and further conducted systematic assessments on the conservation characteristics and strain-specific traits of the well-characterized pigment metabolic pathways across these multiple strains.

The results showed that in yellow/red cocoon strains, the high expression of *CBP* and *Cameo2* genes in the silk gland is a common pattern, confirming the stability of the core pathway across different genetic backgrounds. At the population level, a widespread pattern of variation in the coding region of the *CBP* gene was also verified: all yellow/red cocoon strains carry a complete CDS region, whereas white and green cocoon strains generally exhibit shifts in the start codon and deletions in the N-terminal sequence.

These findings not only verified and further consolidated the established theoretical conclusions at the multi-strain research level but also uncovered the crucial influence of allelic variation on the formation of phenotypic differences among silkworm strains. In turn, this study supplied more accurate genetic target sites and multi-strain resource data for the molecular design breeding of silkworms with specific cocoon color characteristics.

## 2. Materials and Methods

### 2.1. Biological Material and Main Reagents

A total of 14 varieties of silkworms with colorful cocoon from different areas were selected, and they were bred and maintained by the Sericulture Science Research Center, Chinese Academy of Agricultural Sciences. The larvae were reared on mulberry leaves at 28 ± 1 °C and 85% relative humidity during the 1st to 3rd instars, and at 25 ± 1 °C and 70% relative humidity with ventilation from the 4th to 5th instars. The silkworm strains used are listed in Table 1.

Carotenoid and flavonoid standards were provided by Sinopharm Chemical Reagent Co., Ltd. (Shanghai, China). Chemical reagents including ethanol were of analytical grade and were provided by Sangon Biotech Co., Ltd. (Shanghai, China). Reagents for total RNA extraction and reverse transcription were purchased from Vazyme Biotech Co., Ltd. (Nanjing, China). All chemicals for RT-PCR and quantitative real-time PCR (qRT-PCR) were obtained from TransGen Biotech Co., Ltd. (Beijing, China).

### 2.2. Treatment of Experimental Materials

To clarify the patterns of pigment transport and deposition in different silkworm strains, this study designed a systematic sample collection and preservation protocol. Representative yellow/red, green, and white cocoon strains were selected, with two replicate groups per strain (30 larvae per group), and reared individually under standard temperature and humidity conditions.

The first group of silkworms was sampled on the third day of the fifth instar (the peak feeding stage). Following anesthesia on an ice plate, the MG, hemolymph, MSG, and posterior silk gland (PSG) were rapidly dissected. All tissue samples were flash-frozen in liquid nitrogen and stored at −80 °C for subsequent pigment content analysis (e.g., carotenoid and flavonoid) and expression profiling of pigment metabolism-related genes such as *CBP* and *Cameo*2.

The second group of silkworms was reared until cocoon completion, and whole cocoon shells were collected. These samples were primarily used for high-performance liquid chromatography (HPLC) to qualitatively and quantitatively analyze pigments in the cocoon layer, comparing differences in pigment composition and content among the different strains.

### 2.3. Determination of Pigment Compound Content

Collected cocoon shells were cut into pieces and extracted with 70% ethanol using ultrasonic assistance. Silk glands, midguts, and hemolymph were first lyophilized into powder, then extracted with ethanol solution in a 60 °C water bath to obtain pigment sample solutions. For the quantification of multiple carotenoid compounds in 11 silkworm varieties with colorful cocoons (white, yellow, red, and flesh), the extracted pigments were analyzed by high-performance liquid chromatography (HPLC) coupled with a diode array detector. Separation was achieved on a C18 column using a gradient mobile phase system. Individual carotenoids were identified by comparing retention times and absorption spectra with authentic standards and quantified using calibration curves constructed from corresponding standards at their respective maximum absorption wavelengths. For the quantification of multiple flavonoid compounds in 3 silkworm varieties with green cocoons, the extracts were analyzed by HPLC under similar conditions. Individual flavonoids, isorhamnetin, etc.) were identified and quantified using authentic standards, with detection at appropriate wavelengths.

### 2.4. Total RNA Extraction and Reverse Transcription

Total RNA was extracted from various parts of the silk glands and midguts, respectively, which had been stored at −80 °C, using FreeZol Reagent (Vazyme, Nanjing, China). cDNA was then synthesized with the HiScript III 1st Strand cDNA Synthesis Kit (+gDNA wiper).

### 2.5. Quantitative Real-Time PCR

qRT-PCR primers were designed using Premier 6.0 and synthesized by Sangon Biotech (Shanghai, China). The primer sequences are listed in Table 2. qRT-PCR was performed using the ChamQ Blue Universal SYBR qPCR Master Mix kit (Vazyme, Nanjing, China). The reaction mixture (20 μL) contained TBGreen^®^ Premix EX Taq (Takara, Shiga, Japan), 0.5 μL each of forward and reverse primers, 1 μL of template cDNA, and 8 μL of ddH_2_O. The cycling conditions were: 95 °C for 30 s, followed by 40 cycles of 95 °C for 10 s and 60 °C for 30 s, and a final step of 60 °C for 10 min. Each sample was run in three technical replicates. *Actin*3 gene was used as housekeeping gene. Data are presented as the mean ± SEM, and transcript levels were calculated using the 2^−ΔΔCt^ method.

### 2.6. RT-PCR

The expression of *CBP* gene mRNA was detected by PCR using primers listed in Table 2. The 50 μL PCR reaction mixture contained 2 μL of template, 2 μL each of forward and reverse primers, 25 μL of 2×Taq Master Mix, and 19 μL of ddH_2_O. The PCR program was: initial denaturation at 94 °C for 3 min; 35 cycles of 95 °C for 15 s, 60 °C for 15 s, and 72 °C for 60 s; and a final extension at 72 °C for 5 min. Amplified products were separated on a 1.2% agarose gel, and target fragments were identified based on product length and sequencing results.

### 2.7. Sequence Alignment and Gene Structure Analysis

Sequencing of the PCR products was performed by Shanghai Sangon Biotech (Shanghai, China). Sequence alignment was carried out using DNAMAN 9.1.0.2 software.

### 2.8. Statistical Analysis

In the real-time quantitative PCR experiments, statistical significance of differences in gene expression among groups was assessed by one-way analysis of variance (ANOVA). Where ANOVA indicated a significant overall effect (*p* < 0.05), post hoc multiple comparisons were performed using the Least Significant Difference (LSD) test, the Waller-Duncan *t*-test, and Tukey’s s-b (K) method. The results of these comparisons are presented in the figures using letter-notation, which is based primarily on the outcomes of either the Waller-Duncan test or Tukey’s b procedure. According to this notation, groups that do not share a common letter (e.g., “a” vs. “b”) differ significantly (*p* < 0.05), whereas groups sharing at least one common letter (e.g., “a” and “ab”) are not statistically different.

## 3. Results

### 3.1. Cocoon Color and Silk Gland Morphology in Different Colored Cocoon Strains

To visually compare the final cocoon color phenotypes and pigment deposition in the silk glands among different silkworm varieties, we conducted morphological observations and imaging of mature cocoons and the silk glands of fifth-instar larvae from 14 representative silkworm strains.

**Figure 1:**
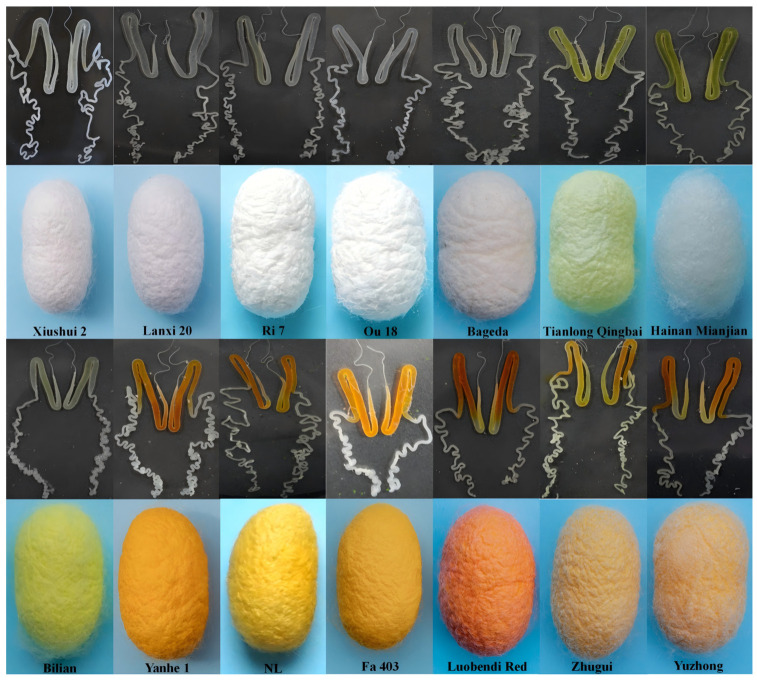
Observation of Cocoon and Silk Gland Morphology in Different Strains.

The results showed that the dissected silk glands also exhibited distinct color differences. The silk glands of yellow/red cocoon strains appeared yellow or orange-yellow, matching their cocoon color. In contrast, the silk glands of green cocoon strains showed a light yellow-green hue, while those of white cocoon strains were mostly white (Figure 1). These visual observations provided direct phenotypic evidence for understanding the cytological basis of cocoon color formation at the tissue level.

### 3.2. Analysis of Differences in Pigment Compound Content Among Different Tissues of the Silkworm

**Figure 2:**
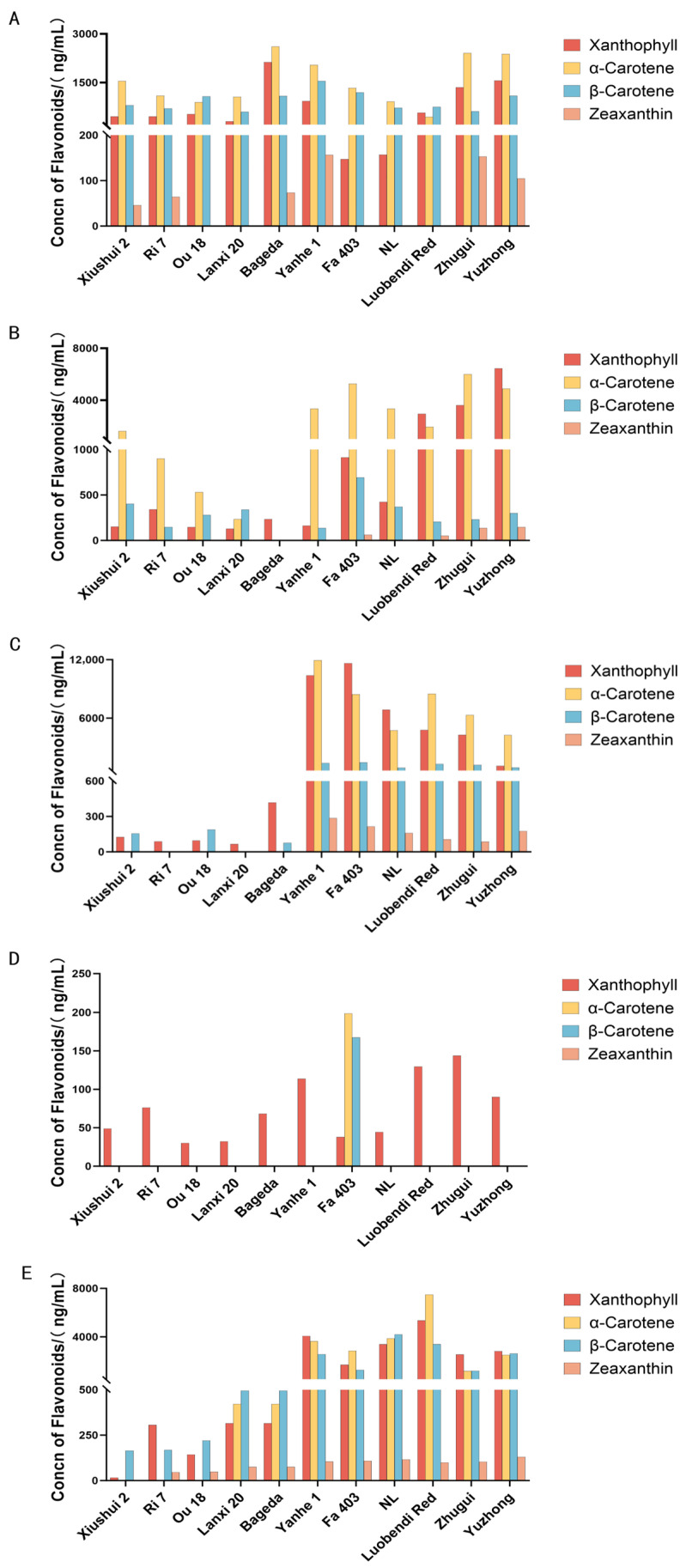
Content of different carotenoid compounds in various tissues of silkworm larvae and in the cocoon. (**A**) Midguts; (**B**) hemolymph; (**C**) middle silk glands; (**D**) posterior silk glands; (**E**) cocoon shell. The types of carotenoid compounds are listed in the upper right corner of the figure.

In this study, the pigment compound content among different tissues of the silkworm was determined firstly. High-performance liquid chromatography (HPLC) was used to systematically determine the composition and content of carotenoids in different tissues of 11 silkworm strains on the third day of the fifth instar. The results showed that the total carotenoid content in the MG of white cocoon strains was generally lower than that in yellow/red cocoon strains (Figure 2A). Notably, however, the xanthophyll content in the MG of the yellow-hemolymph white cocoon strain Bageda reached 2127.91 ng/mL, about 10 times higher than that of other white-hemolymph white cocoon strains, and its β-carotene level was 797.01 ng/mL, indicating a strong capacity for carotenoid absorption and accumulation in this strain.

Carotenoid content in the hemolymph is often regarded as a key indicator of pigment transport to the silk gland. In all white cocoon strains, total carotenoids in the hemolymph were significantly lower than in yellow/red cocoon strains, suggesting possible systematic differences in pigment transport efficiency among different strains (Figure 2B).

The MSG is the main site of carotenoid deposition, and its pigment content directly affects cocoon color. In yellow/red cocoon strains, xanthophyll and α-carotene accumulated in the MSG reached levels as high as 10,377.93 ng/mL and 11,929.43 ng/mL, respectively, while β-carotene also remained at relatively high levels (Figure 2C). In contrast, the PSG exhibited very low carotenoid content across all strains, with most compounds undetectable, further supporting the conclusion that carotenoids are primarily deposited in the sericin layer of the MSG (Figure 2D).

The composition of carotenoids in the cocoon layer showed clear strain-specific patterns. For instance, the yellow-hemolymph yellow cocoon strain Yanhe 1 was dominated by xanthophyll and α-carotene, while the red cocoon strain Luobendi Red was primarily characterized by α-carotene, followed by xanthophyll, consistent with the general pattern of red cocoon strains. In flesh-colored cocoon strains, similar proportions of α-and β-carotene formed the material basis for their light color phenotype (Figure 2E).

Additionally, some strains exhibited specific carotenoid distribution patterns. For example, zeaxanthin was not detected in any tissue of strains Ou18 and Lanxi20, except for trace amounts in the midgut. Similarly, strains Fa403, NL, and Luobendi Red accumulated almost no zeaxanthin in any tissue. These differences provide important clues for further elucidating the metabolic regulatory network underlying cocoon color formation.

**Figure 3:**
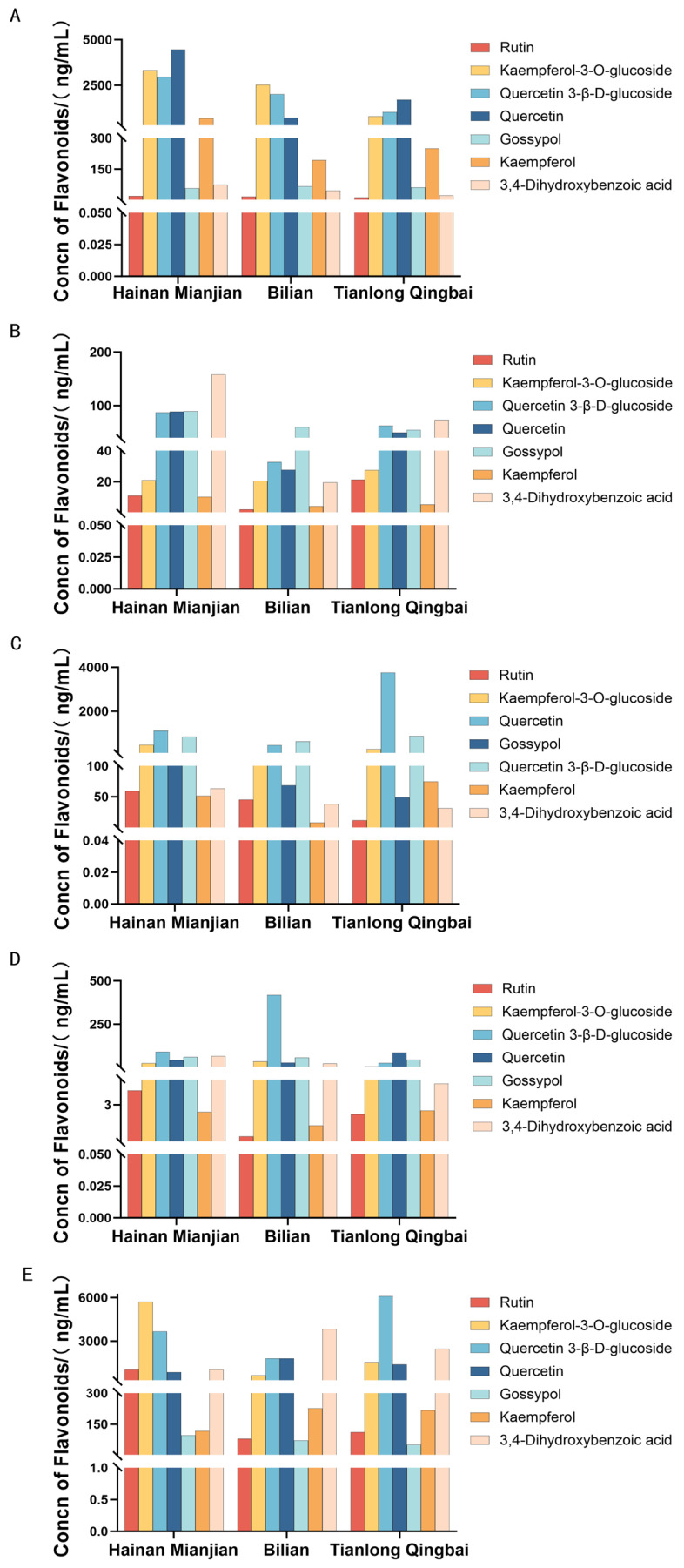
Content of different flavonoid compounds in various tissues of the silkworm and in the cocoon. (**A**) Midguts; (**B**) hemolymph; (**C**) middle silk glands; (**D**) posterior silk glands; (**E**) cocoon shell. The types of different flavonoid compounds are listed in the upper right corner of the figure.

Systematic quantification of seven flavonoid compounds in various tissues and cocoon shells of three representative green cocoon silkworm strains revealed that total flavonoid content was significantly accumulated in the silk gland (Figure 3), especially in the MSG and in the cocoon shell. Their concentrations were generally higher than those in the MG and hemolymph, indicating that the silk gland is the key site for flavonoid deposition, with the MSG playing a central role in the directional enrichment of pigments.

Further analysis showed distinct flavonoid profiles among the different green cocoon strains. The cocoon shell of Hainan Mianjian was dominated by glycosylated forms such as kaempferol-3-O-glucoside and quercetin-3-β-D-glucoside, with low levels of aglycones, suggesting that flavonoid metabolism in this strain may be primarily driven by glycosylation. In contrast, the cocoon shell of Bilian accumulated substantial amounts of 3,4-dihydroxybenzoic acid and quercetin, with a lower proportion of glycosides, indicating potentially stronger flavonoid catabolism or deglycosylation activity. The cocoon shell of Tianlong Qingbai showed high enrichment of quercetin-3-β-D-glucoside along with elevated quercetin content, reflecting a unique balance between glycoside storage and aglycone accumulation in this strain.

### 3.3. Analysis of the Differential Expression of Cocoon Color-Related Genes in Various Tissues Among Different Silkworm Strains

**Figure 4:**
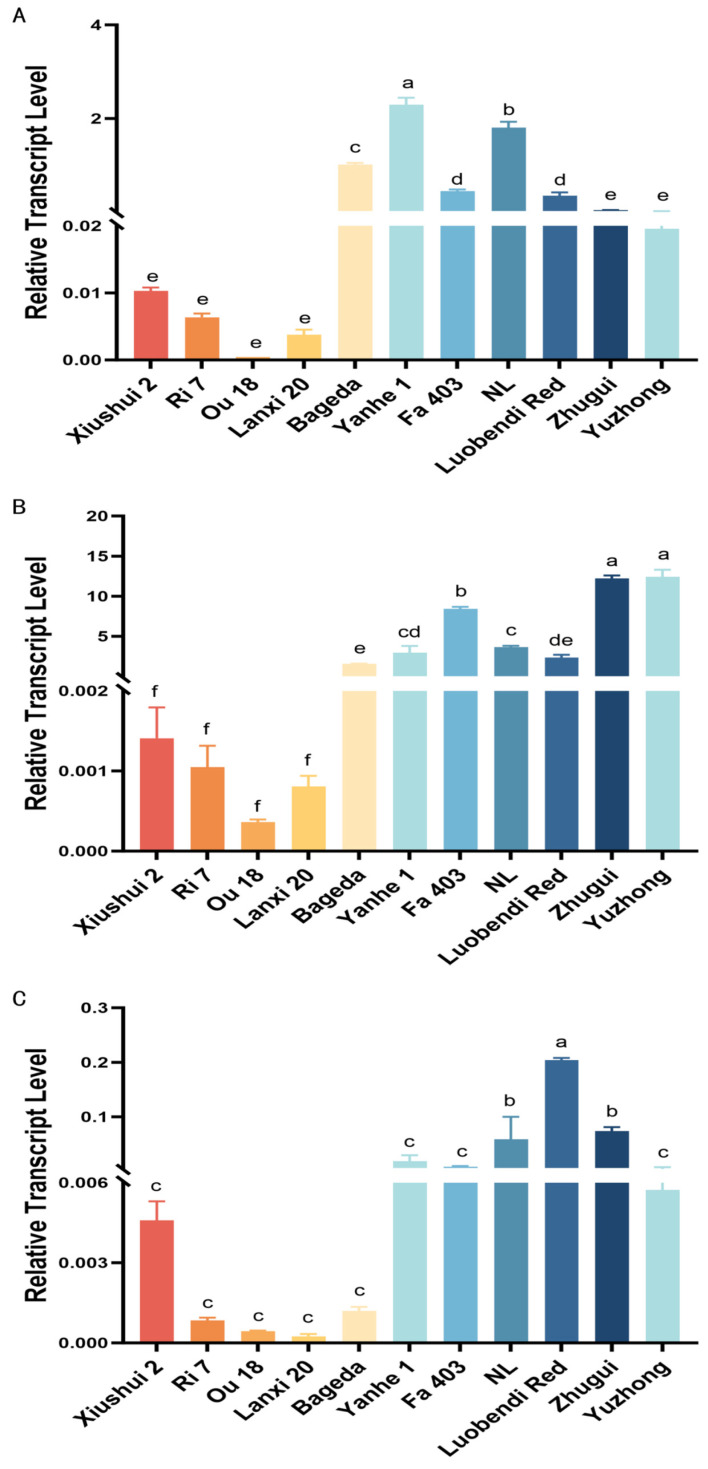
Analysis of the relative expression level of the *CBP* gene in various tissues among different silkworm strains. (**A**) Midguts; (**B**) middle silk glands; (**C**) posterior silk glands. Different lowercase letters (a, b, c, d, e, f) indicate statistically significant differences among groups (*p* < 0.05). Groups that share a common letter (e.g., both labeled “a”) or that have overlapping letters (e.g., “ab” and “a”) are not significantly different; groups that do not share any common letter (e.g., “a” vs. “b”) differ significantly (*p* < 0.05).

To explore the reason of differential pigment compound content among different tissues of the silkworm, the expression levels of cocoon color-related genes were also measured. The relative expression levels of the carotenoid binding protein (*CBP*) gene in the MG, MSG and PSG of 11 silkworm strains with different cocoon colors were examined systematically by using quantitative real-time PCR. Tissue-specific analysis results showed that the expression level of *CBP* was highest in the MSG, followed by the MG and PSG. Particularly in yellow/red cocoon strains, its expression level in the MSG was significantly higher than in other tissues. For example, in the yellow cocoon strain Fa403, the expression level in the MSG was about 18.7-fold that in its MG. Similarly, in the red cocoon strain Luobendi Red, MSG expression reached 6.7-fold that in the MG, further supporting the key role of the MSG in carotenoid deposition (Figure 4).

Comparison among different strains revealed that the relative expression level of *CBP* in all tissues of white cocoon strains was generally below 0.01, whereas yellow/red strains showed higher expression in the MSG, with two flesh-colored strains reaching levels of 12.19 and 12.41. Notably, the yellow-hemolymph white cocoon strain Bageda, although producing white cocoons, exhibited CBP expression levels of 1.01 in the MG and 1.55 in the MSG. These values were significantly higher than those in other white cocoon strains and even exceeded some yellow cocoon strains, suggesting a strong potential for carotenoid absorption and transport in this strain, and indicating that its cocoon color formation may be regulated downstream in metabolism or deposition.

**Figure 5:**
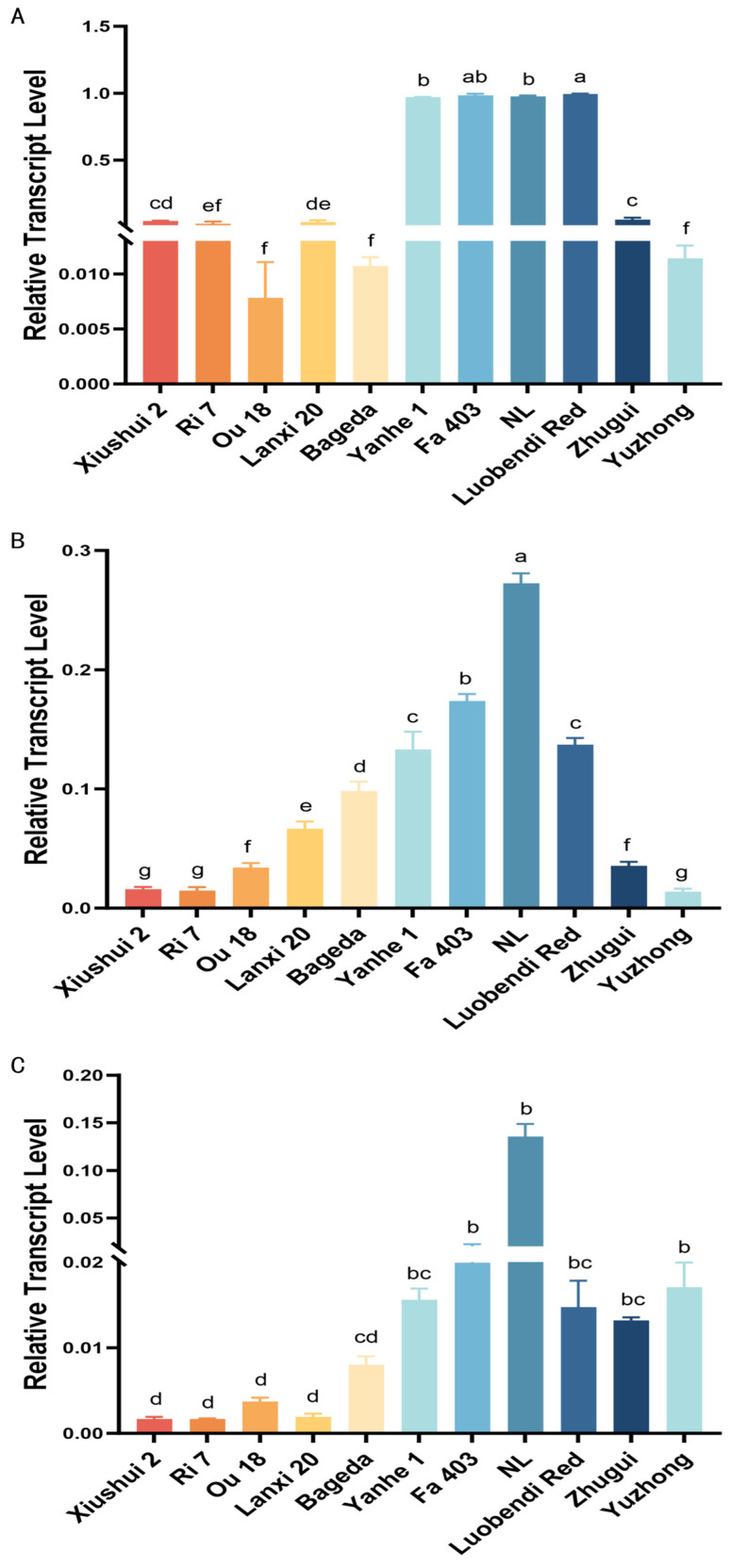
Analysis of the relative expression level of the *Cameo*2 gene in various tissues among different silkworm strains. (**A**) Midguts; (**B**) middle silk glands; (**C**) posterior silk glands. Different lowercase letters (a, b, c, d, e, f, g) indicate statistically significant differences among groups (*p* < 0.05). Groups that share a common letter (e.g., both labeled “a”) or that have overlapping letters (e.g., “ab” and “a”) are not significantly different; groups that do not share any common letter (e.g., “a” vs. “b”) differ significantly (*p* < 0.05).

Quantitative analysis of the *Cameo*2 gene expression pattern across 11 silkworm strains revealed that in the MG, yellow and red cocoon strains maintained relatively high expression levels, whereas flesh-colored and white cocoon strains generally showed levels below 0.06. This suggested that the high expression of *Cameo*2 in MG may be directly associated with the carotenoid accumulation capacity of yellow/red cocoon strains (Figure 5A).

In silk glands, strain NL exhibited the highest *Cameo*2 expression in both the MSG and PSG, further indicating that differences in pigment deposition among strains have a gene expression basis (Figure 5B,C). Moreover, two flesh-colored strains showed even lower expression in the MSG than some white cocoon strains, implying that the role of *Cameo*2 in flesh-colored cocoon formation may differ from that in other yellow/red phenotypes. The specific regulatory mechanism involved requires further investigation.

Analysis of *SCRB*15 gene expression revealed that its transcript abundance was significantly lower in the MG than in the silk gland, indicating clear tissue-specific expression. Notably, in the white cocoon strains Xiushui 2 and Ri 7, the transcript levels of *SCRB*15 across various tissues were not only generally higher than in other white cocoon strains but also exceeded those in some yellow cocoon strains. This suggested that although these strains exhibit relatively active mRNA transcription of *SCRB*15, their final cocoon color does not display the corresponding pigmentation. This discrepancy may result from lower translational efficiency after transcription or from reduced stability and rapid degradation of the synthesized SCRB15 protein, preventing the accumulation of sufficient functional protein product to complete pigment deposition.

In contrast, the red cocoon strain Luobendi Red and the flesh-colored strains Zhugui and Yuzhong maintained significantly higher *SCRB*15 transcript levels in all tissues compared with other strains. This result aligns with the known role of *SCRB*15 as the product of *F* gene (flesh-colored cocoon gene) and directly confirms the central function of this gene in determining the flesh-colored phenotype.

**Figure 6:**
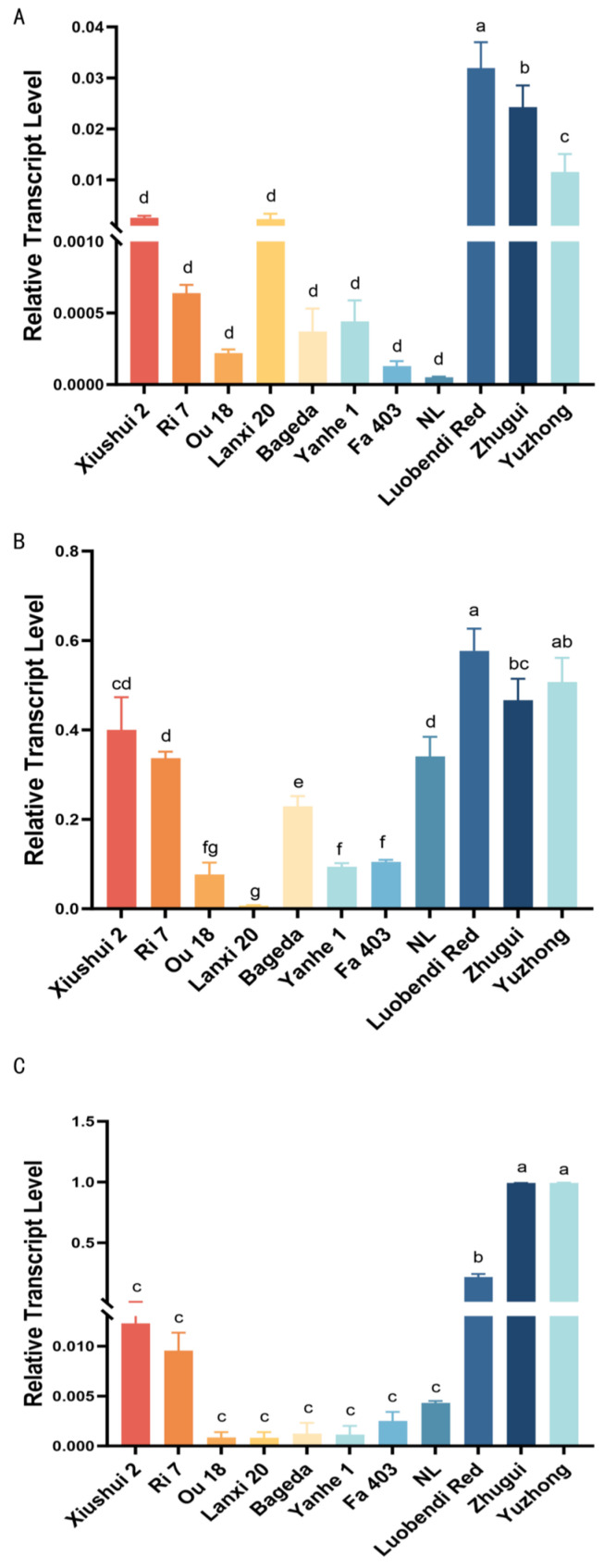
Analysis of the relative expression level of the *SCRB*15 gene in various tissues among different silkworm strains. (**A**) Midguts; (**B**) middle silk glands; (**C**) posterior silk glands. Different lowercase letters (a, b, c, d, e, f, g) indicate statistically significant differences among groups (*p* < 0.05). Groups that share a common letter (e.g., both labeled “a”) or that have overlapping letters (e.g., “ab” and “a”) are not significantly different; groups that do not share any common letter (e.g., “a” vs. “b”) differ significantly (*p* < 0.05).

Interestingly, a high expression of *SCRB*15 was also observed in the red cocoon strain, implying that the formation of red phenotype may similarly rely on high-flux transport of β-carotene or involve allele-specific regulation of *SCRB*15. This finding expanded the understanding of *SCRB*15 function in cocoon color formation, indicating that its elevated expression may be a common molecular feature among certain yellow/red cocoon strains (including some red and flesh-colored varieties) that primarily use β-carotene as the major pigment (Figure 6).

**Figure 7:**
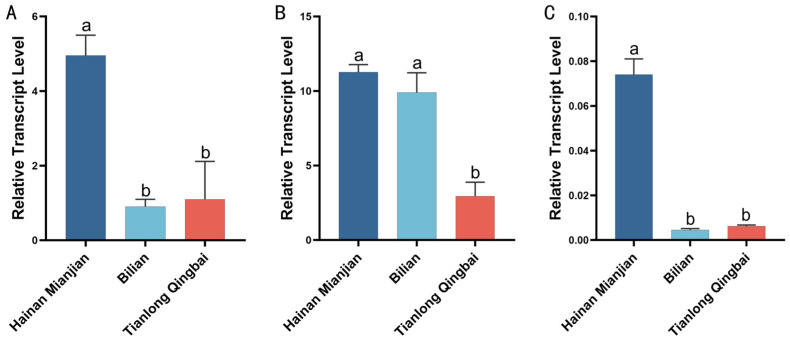
Analysis of the relative expression level of the *UGT*86 gene in various tissues among different silkworm strains. (**A**) Midguts; (**B**) middle silk glands; (**C**) posterior silk glands. Different lowercase letters (a, b) indicate statistically significant differences among groups (*p* < 0.05). Groups that share a common letter (e.g., both labeled “a”) or that have overlapping letters (e.g., “ab” and “a”) are not significantly different; groups that do not share any common letter (e.g., “a” vs. “b”) differ significantly (*p* < 0.05).

Analysis of *UGT* gene expression in three green cocoon silkworm strains (Hainan Mianjian, Bilian, and Tianlong Qingbai) showed that the highest transcript levels occurred in the MSG across all strains. This pattern aligns with the central role of the UGT86 protein in the glycosylation of flavonoids and cocoon color formation. Expression in the MG was relatively low, with Hainan Mianjian showing the highest level and Bilian the lowest (Figure 7A).

In the MSG, Hainan Mianjian exhibited significantly higher expression than the other two strains, indicating notable differences in flavonoid glycosylation capacity among green cocoon varieties (Figure 7B). In the PSG, expression levels were very low in all strains, with the highest value nearly 20-fold greater than the lowest (Figure 7C).

### 3.4. Sequence Comparison of the CBP Gene Among Different Strains

**Figure 8:**
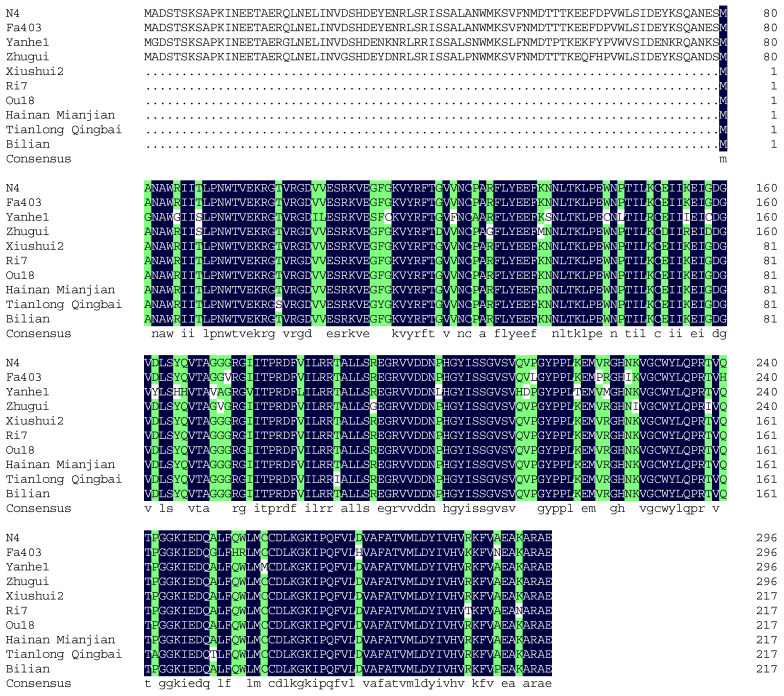
Amino acid sequence alignment of the CBP protein among different cocoon-color varieties of *Bombyx mori*. N4, Fa 403, Yanhe 1 belong to the yellow cocoon strains; Zhugui belongs to the flesh-colored cocoon strain; The other belong to white cocoon strains. Blue: conserved residues (identical across all varieties). Green: variable residues (differences among varieties).

The differences of sequences of *CBP* genes among different colored cocoon strains are still unknown. Using the known CDS sequence of the *CBP* gene, an upstream primer was designed to perform PCR amplification and sequencing analysis on nine silkworm strains: Yanhe1, Fa403, and Zhugui (yellow/red cocoon strains); Xiushui2, Ri7, and Ou18 (white cocoon strains); and Hainan Mianjian, Bilian, and Tianlong Qingbai (green cocoon strains) (Figure 8).

In the yellow/red cocoon strains, the amplified coding sequence contained the start codon at the conventional position. The sequence was 894 bp in length, encoding 296 amino acids, consistent with the fully annotated reference sequence N4 from the NCBI database.

In contrast, the white and green cocoon strains showed a shifted transcription start site. Sequencing results revealed that the start codon corresponds to a position 208 bp downstream within the coding region of the yellow/red strains (and the reference sequence). From the start codon onward, the coding sequence of the white and green strains matched the 657 bp region (positions 209-894) of the yellow/red strain coding sequence, indicating that it likely encodes a truncated CBP protein of 217 amino acids with a partial N-terminal deletion.

## 4. Discussion

Owing to their distinctive value in the development of ecological textiles and functional materials, natural-colored silkworm cocoons have emerged as a vital development direction for the modern sericultural industry [24]. This research systematically uncovered the inherent patterns of pigment accumulation and distribution that underpin the different cocoon color phenotypes in silkworms. A central finding of this study is that pigment deposition shows striking tissue-specific and strain-specific features. The middle silk gland is confirmed as the key tissue for the deposition of both carotenoid and flavonoid pigments, which aligns with its functional role in sericin synthesis, as the sericin layer serves as the essential binding matrix for various pigments [25]. The disparities in pigment accumulation capabilities of key tissues among different silkworm strains directly shape their final cocoon color traits. For example, silkworm strains producing yellow or red cocoons can efficiently accumulate carotenoids in the middle silk gland, while green cocoon strains exhibit specific enrichment of flavonoids in this tissue.

Of particular interest is that the Bageda strain, which possesses yellow hemolymph but produces white cocoons, displayed a distinctive metabolic profile. Although its midgut exhibited strong ability to absorb carotenoid substances, such pigments could not be efficiently stored in the cocoon shell. This inconsistent result implies that the obstruction of cocoon pigmentation may occur during the transfer from midgut to silk gland or within the silk gland deposition step, rather than only at the absorption stage. Such results offer meaningful reference for subsequent investigations focused on the functional mechanisms of carrier proteins.

In addition, obvious differences in the pigment composition of the cocoon shell including the proportion of each individual carotenoid and the ratio between flavonoid glycosides and aglycones demonstrated that the ultimate cocoon color is determined not only by the total pigment content but also by the strain-specific distribution of metabolic flux. For example, differences in the glycosylation degree of flavonoids among green cocoon strains directly reflect variations in the activity of modifying enzymes like UDP-glycosyltransferases (UGTs). Collectively, these research results clarified the chemical foundation of cocoon color diversity at the metabolite level, connecting phenotypic differences to the underlying enzymatic activities and gene expression regulatory processes, and laying a theoretical groundwork for the targeted molecular improvement of cocoon color characteristics.

Expression analysis of key pigment transport and modification genes provided transcription-level support and refinement for the aforementioned metabolic patterns. The high expression of *Cameo*2 in the midgut and the extremely high expression of *CBP* in the middle silk gland of yellow/red cocoon strains validated the classic “midgut absorption-silk gland deposition” transport pathway of carotenoids across multiple strains. The elevated expression of *SCRB*15 in red and flesh cocoon strains further confirmed its role as a core molecular marker determining the β-carotene deposition phenotype. For green cocoon strains, the specific high expression of *UGT*86 in the silk gland and its variation among strains offered a direct transcriptional explanation for the diversity in flavonoid glycosylation levels observed in different green cocoons. It is noteworthy, however, that while expression patterns of these key genes have previously helped establish genotype-phenotype associations in specific strains, the construction of a silkworm super-pangenome and systematic analysis of more germplasm resources allowed this study to test these patterns across a broader range of strains, revealing exceptions where gene expression levels did not fully correlate with the final cocoon color phenotype. This phenomenon suggested that cocoon color formation may be influenced by multi-layered factors such as post-transcriptional regulation, epigenetic modifications, protein interaction networks, or as-yet-unidentified rare variants. The existence of these exceptions highlights the complexity of the genetic basis underlying cocoon color traits in silkworms and indicates that future work should integrate multi-omics data and functional experiments to unravel the complete regulatory network at a finer molecular level.

It is worth emphasizing that when verifying these expression patterns across a wider range of silkworm strains, special cases were also found in which gene expression levels did not fully match the final phenotypic traits. As a specific illustration, the Baghdad strain, which has yellow hemolymph but forms white cocoons, exhibits relatively high *CBP* gene transcript abundance; additionally, certain white cocoon strains show substantial *SCRB*15 transcriptional activity, yet their cocoons do not present the corresponding coloration phenotype. These findings strongly implied that apart from the transcriptional regulatory mechanisms analyzed in this study, critical post-transcriptional regulatory processes-such as translational efficiency, protein stability, or post-translational modification events-may result in the phenomenon of “high transcription levels without corresponding phenotypic manifestation”. This discovery offers a novel research perspective for deciphering the complete regulatory network of silkworm cocoon color formation and also indicates that subsequent studies should go beyond transcriptome analysis to explore the precise regulatory mechanisms at the post-transcriptional and protein levels.

Variations in gene sequences have a direct impact on the functional properties of the proteins they encode [26]. The present research identified an essential structural distinction in the coding sequence (CDS) of the *CBP* gene between silkworm strains with yellow/red cocoons and those with white/green cocoons. Strains producing yellow or red cocoons harbor a complete full-length CDS (894 bp), while white and green cocoon strains showed a displacement in the transcription start site. This displacement is predicted to generate a truncated protein that lacks a portion of the N-terminal domain. Such structural variation is likely to weaken the truncated protein’s capacity to bind or transport carotenoid pigments, thereby genetically restricting these strains from forming yellow or red cocoons. This research provided a DNA-level interpretation for the genetic stability of specific cocoon color phenotypes and supplies a direct genetic target for marker-assisted selection (MAS) in the breeding of silkworms with desired cocoon color traits.

By conducting systematic analysis on a variety of silkworm strains, this research verified the core rules governing pigment metabolism and deposition at the population level, while supplementing new supporting evidence to the established theoretical system. The study confirmed that the middle silk gland (MSG) serves as the core tissue for pigment deposition, and the expression profiles of key genes (*CBP*, *Cameo*2, *SCRB*15, and *UGT*86) are closely matched with pigment distribution characteristics. This finding further supports the classic “absorption-transport-deposition” metabolic pathway across different silkworm strains. Meanwhile, this research uncovers more intricate regulatory mechanisms within a wider genetic background. The inconsistency between gene expression levels and the ultimate cocoon color observed in certain strains indicates the existence of post-transcriptional or translational regulatory junctions. Furthermore, structural differences in the coding region of the *CBP* gene across silkworm strains with different cocoon colors offers an explanation at the DNA sequence level for the genetic stability of specific phenotypic variations. These results not only reinforced the understanding of silkworm cocoon color formation but also offered more comprehensive strain resources and a theoretical basis for subsequent trait improvement using molecular markers.

Cocoon color formation is a complex physiological process involving the coordinated regulation of multiple metabolic pathways. In this study, we focused primarily on the carotenoid metabolic pathway and the expression profiles of related genes—such as *CBP*, *Cameo*2, and *UGT*86—across silkworm strains with different cocoon colors. Our results highlight the critical role of carotenoid deposition mechanisms in determining cocoon color variation. However, the final pigmentation phenotype is likely shaped not only by carotenoids but also by melanin synthesis pathways. In insects, the prophenoloxidase (PPO) activation cascade is central to melanin production, where a series of serine proteases convert PPO into active phenoloxidase (PO), which then catalyzes the oxidation of phenols to quinones, ultimately leading to melanin formation. Notably, Serpin-4 functions as a negative regulator of this cascade by targeting serine proteases such as HP1, SP2, and SP6, thereby suppressing PPO activation and modulating melanin deposition [27]. Additionally, heme peroxidases (HPXs) are widely involved in extracellular matrix stabilization, immune responses, and tissue remodeling in insects. For instance, in Anopheles mosquitoes, HPX15 facilitates protein cross-linking to form a midgut epithelial barrier, thereby influencing immune balance and indirectly affecting Plasmodium development [28]. Although the direct involvement of HPXs in silkworm cocoon pigmentation remains unexplored, their capacity to catalyze protein cross-linking and oxidation reactions raises the possibility that they may contribute to matrix modification within the silk gland or interact with melanin synthesis pathways. In the silkworm, PO activity varies among strains and correlates with cuticular pigmentation. Thus, while our findings underscore the predominant role of carotenoid-associated genes in cocoon color differentiation, the potential contribution of melanin-related pathways, including regulatory factors such as Serpin-4, and the possible involvement of heme peroxidases warrant further investigation. Future studies integrating carotenoid and melanin pathways, along with other oxidative enzyme systems, will be essential for a more comprehensive understanding of the molecular network underlying cocoon pigmentation.

## Figures and Tables

**Table 1:** Experimental Silkworm Varieties and Their Cocoon Color Classification.

Name	Cocoon Color	Strain	Name	Cocoon Color	Strain
Xiushui 2	White	Chinese race	Bilian	Green	Chinese race
Lanxi 20	White	Chinese race	Yanhe 1	Yellow	Chinese race
Ri 7	White	Japanese race	NL	Yellow	Japanese race
Ou 18	White	European race	Fa 403	Yellow	European race
Bageda	White	European race	Luobendi Red	Red	European race
Tianlong Qingbai	Green	Japanese race	Zhugui	Flesh	Chinese race
Hainan Mianjian	Green	Chinese race	Yuzhong	Flesh	European race

**Table 2:** Primer sequences for PCR.

Reaction Type	Gene Name	Primer Sequence (5′-3′)	Length of Product (bp)
qRT-PCR	*Actin3*	F: CGGCTACTCGTTCACTACC R: AGCAATTCACACAAGGCAGT	147
	*CBP*	F: CGAAAAGCGCGCCAAAAATC R: GAACTCCTCCTTGGTCGTGG	170
	*Cameo2*	F: GGAGTTTGTGGAGAGTGGGG R: ACTCGCCCTTACAGAAGCAC	119
	*SCRB15*	F: TGTCTTCTGTCGCTCAATCCT R: CCCTCTTGAGGCGGAGAAACC	134
	*UGT86*	F: TTGCACCCATAACGTCTCCC R: ACGGCTTTGGATGGATGGTT	172
PCR	*CBP*	F: CGAAAAGCGCGCCAAAAATC R: GCTTCACGGCGCAGAAATAG	995

## Data Availability

The original contributions presented in this study are included in the article. Further inquiries can be directed to the corresponding authors.
